# Efetividade do Programa Parto Adequado na diminuição das taxas de cesárea de maternidades privadas no Município de São Paulo, Brasil

**DOI:** 10.1590/0102-311XPT216623

**Published:** 2024-11-11

**Authors:** Andrea Silveira De Queiroz Campos, Daphne Rattner, Carmen Simone Grilo Diniz

**Affiliations:** 1 Faculdade de Saúde Pública, Universidade de São Paulo, São Paulo, Brasil.; 2 Faculdade de Ciências da Saúde, Universidade de Brasília, Brasília, Brasil.

**Keywords:** Parto, Cesárea, Saúde Materno-infantil, Políticas de Saúde, Estudos de Avaliação, Parturition, Cesarean Section, Maternal and Child Health, Health Policies, Evaluation Studies, Parto, Cesárea, Salud Materno-infantil, Políticas de Salud, Estudios de Evaluación

## Abstract

A cesárea é uma intervenção que salva vidas, mas seu uso sem indicação obstétrica está relacionado a complicações a curto e longo prazo. O Brasil é conhecido internacionalmente por suas altas taxas desse procedimento, ainda mais elevadas no setor privado. Para reverter esse problema, a Agência Nacional de Saúde Suplementar lançou o Programa Parto Adequado, e este estudo tem como objetivo analisar a sua efetividade. Neste estudo retrospectivo, incluímos a totalidade de nascimentos em maternidades privadas ocorridos entre 2014 e 2019 para comparar a evolução das taxas de cesárea de hospitais participantes e não participantes do projeto, a partir das bases de dados públicas do Sistema de Informações sobre Nascidos Vivos (SINASC). Foram analisados 277.747 nascimentos, sendo observada uma redução na taxa de cesárea nos dois grupos, mais acentuada entre os hospitais participantes. Não foi observada redução da chance de cesárea antes do lançamento do programa (2014), mas notou-se uma tendência constante de redução após 2014, até se tornar significativa em 2018. Esse resultado ocorreu de forma independente das variáveis demográficas, maternas e dos grupos de Robson. A taxa de cesárea dos hospitais participantes do programa foi de 83,8% para 72,3% (intervalo de 95% de confiança - IC95%: 71,7-72,9). Apesar da redução, permanece bem acima das taxas esperadas de acordo com a ferramenta *c-model*, que seria de 45,2% (IC95%: 33,9-56,5) para essa população. Os resultados deste estudo mostraram que uma política pública bem conduzida com o envolvimento de instituições privadas pode mudar o cenário da atenção ao parto e ao nascimento, promovendo a redução das altas taxas de cesárea.

## Abstract (en)

Cesarean section is a life-saving intervention, but its use without obstetric indication leads to short- and long-term complications. Brazil is internationally known for its high cesarean rates, especially in the private sector. To reverse this problem, the Brazilian National Regulatory for Private Health Insurance and Plans launched the Adequate Childbirth Program, and this retrospective study aims to analyze its effectiveness. We included the total of births performed in private maternity hospitals between 2014 and 2019 registered on the Brazilian Information System on Live Births (SINASC) database to compare the evolution of cesarean section rates in hospitals participating and not participating in the project. A total of 277,747 births were analyzed, showing a reduction in the cesarean section rate in both groups but more pronounced among the participant institutions. No reduction in cesarean performance was observed before the program launch (2014), but there was a constant downward trend after 2014, until it became significant in 2018. This result occurred independently of demographic, maternal, and Robson group variables. The cesarean section rate of hospitals participating in the program went from 83.8% to 72.3% (95% confidence interval - 95%CI: 71.7-72.9). Despite the reduction, it remains well above the expected rates according to the c-model tool, which would be 45.2% (95%CI: 33.9-56.5) for this population. Results showed that a well-conducted public policy together with private institutions can change the scenario of labor and birth care, reducing high cesarean section rates.

## Abstract (es)

La cesárea es una intervención que salva vidas, pero su uso sin indicación obstétrica está relacionado con complicaciones a corto y largo plazo. Brasil es conocido internacionalmente por sus elevadas tasas de este procedimiento, que son aún mayores en el sector privado. Para revertir este problema, la Agencia Nacional de Salud Complementaria lanzó el Programa Parto Adecuado, y este estudio tiene como objetivo analizar su efectividad. En este estudio retrospectivo, incluimos todos los nacimientos en maternidades privadas que ocurrieron entre el 2014 y el 2019 para comparar la evolución de las tasas de cesáreas en hospitales participantes y no participantes del proyecto, con base en las bases de datos públicas del Sistema de Informacines de Nacidos Vivos (SINASC). Se analizaron 277.747 nacimientos, y se observó una reducción en la tasa de cesáreas en ambos grupos, más pronunciada entre las participantes. No se observó ninguna reducción en las posibilidades de cesárea antes del lanzamiento del programa (2014), pero se observó una tendencia constante de reducción después del 2014, hasta que se volvió significativa en el 2018. Este resultado se produjo de forma independiente de las variables demográficas, maternas y de los grupos de Robson. La tasa de cesáreas de los hospitales participantes en el programa pasó del 83,8% al 72,3% (intervalo de 95% de confianza - IC95%: 71,7-72,9). A pesar de la reducción, se mantiene muy por encima de las tasas esperadas según la herramienta del *c-model*, que sería del 45,2% (IC95%: 33,9-56,5) para esta población. Los resultados de este estudio mostraron que una política pública bien conducida con la participación de instituciones privadas puede cambiar el escenario de la atención al parto y al nacimiento, promoviendo la reducción de las elevadas tasas de cesárea.

## Introdução

A cesárea é uma cirurgia que salva vidas quando existem problemas na gravidez ou no parto; porém, seu uso indiscriminado e sem indicação clínica está relacionado a complicações a curto e longo prazo, além de aumento nos gastos com a saúde ^1^ e, por esse motivo, estratégias para sua utilização de forma adequada têm sido estudadas pela Organização Mundial da Saúde (OMS) e por países com altas taxas desse procedimento ^2^.

A partir de uma reunião promovida pela OMS em 1985 em Fortaleza, analisando dados de países do norte europeu que apresentavam ótimos resultados perinatais, foi considerado que a taxa populacional ideal de cesárea estaria entre 10% e 15%. Essa taxa foi reafirmada em estudos mais recentes; porém, esse valor é questionado por países com taxas mais altas de cesárea. Vários estudos têm sido desenvolvidos para a revisão dessas taxas, sendo tal tarefa considerada um grande desafio devido à heterogeneidade das populações e das estruturas dos serviços de saúde ^2^.

Estudos populacionais mais recentes concluíram que taxas de cesárea menores que 10% estão relacionadas ao aumento de mortalidade materna e associadas com a falta de acesso aos serviços de saúde. Quando taxas de cesárea são maiores que 10% e aumentam até 30%, não se observam efeitos sobre as taxas de mortalidade ^2^, mas taxas maiores que as clinicamente necessárias podem apresentar mais riscos do que benefícios para a população ^3^.

Para auxiliar tais estudos, a OMS recomenda, desde 2015, que seja adotada a classificação de Robson, que classifica as mulheres no momento da admissão para o parto de acordo com cinco características obstétricas (paridade; início do trabalho de parto espontâneo ou induzido; idade gestacional; apresentação fetal; e número de fetos). Dessa forma, pode ser feita uma avaliação das taxas de cesáreas entre esses grupos, o que permite a comparação de resultados maternos e perinatais em um mesmo serviço ao longo do tempo e entre diferentes serviços de saúde, recomendando que tais dados sejam divulgados publicamente sempre que possível. O objetivo da adoção dessa classificação é otimizar o uso das cesáreas por meio da identificação dos grupos mais relevantes de acordo com o risco epidemiológico para procedimento cirúrgico, avaliar as estratégias de melhoria na qualidade do atendimento obstétrico e na coleta dos dados ^2^. O Brasil foi pioneiro em incluir na ficha do Sistema de Informações sobre Nascidos Vivos (SINASC), desde 2012, todas as variáveis necessárias para a classificação de Robson.

Com o objetivo de estabelecer uma referência global de taxa de cesárea de acordo com o serviço de saúde, a OMS desenvolveu uma ferramenta chamada *c-model*, que utiliza modelos matemáticos que consideram características obstétricas utilizadas na classificação de Robson e outras características clínicas para avaliar a taxa de cesárea esperada no serviço analisado ^4^. Tal ferramenta está disponibilizada na internet e é recomendada pela OMS ^5^.

No ano de 2019, o Brasil ocupava o segundo lugar no ranking mundial, com uma taxa geral de cesárea de 57%, sendo tal taxa ainda maior quando considerado apenas o setor privado. De acordo com relatório do Ministério da Saúde, o setor privado foi o responsável pelo financiamento de 287.166 ^6^ dos 2.849.146 partos ocorridos no Brasil naquele ano, o que corresponde a 10% dos nascimentos no país ^7^. Nesse setor, a proporção de nascimentos por cesárea foi de 85% naquele ano ^7^.

São Paulo é a mais populosa cidade brasileira, com um índice de desenvolvimento humano (IDH) de 0,805 (classificado como muito alto) e em estágio IV de transição obstétrica (coeficiente de mortalidade materna baixo, principalmente por causas indiretas e por baixa fertilidade, sendo adotadas como estratégias de melhoria a qualificação da assistência e a diminuição do excesso de intervenções no parto) ^8^. É também uma das cidades com maior cobertura de saúde privada do Brasil, sendo de 43% (intervalo de 95% de confiança - IC95%: 40-47), de acordo com o último inquérito de saúde da cidade de São Paulo, realizado em 2015 ^9^. Dados do *Painel de Monitoramento de Nascidos Vivos* ^7^ segundo Classificação de Risco Epidemiológico (grupos de Robson) do SINASC, apontam que em uma seleção dos hospitais exclusivamente privados (sem leitos contratados pelo Sistema Único de Saúde - SUS), em 2019, a mediana da taxa de cesárea da cidade foi de 80% (quartil 1 a 3: Q1-Q3: 73-86).

Com o objetivo de melhorar a qualidade na assistência obstétrica por meio da redução da taxa de cesáreas desnecessárias ^10^, foi lançado, em 2015, o Programa Parto Adequado (PPA) ^10^ ^,^ ^11^. Esse foi o resultado de uma ação civil pública ocorrida no Estado de São Paulo após a denúncia da rede de mulheres Parto do Princípio ^12^ acerca das elevadas taxas de cesárea encontradas no setor privado. A proposta do PPA incluiu ações educativas, treinamento e conscientização de profissionais de saúde e de usuárias, tendo sido implantado em três fases. A fase 1, fase piloto, entre 2015 e 2016, contando com 35 hospitais; a fase 2, a partir de 2017, com ampliação para 108 hospitais; e a fase 3, iniciada em 2019, com o objetivo de disseminar as estratégias de melhoria da qualidade da atenção do parto e nascimento em grande escala ^10^ ^,^ ^11^ ^,^ ^13^ ^,^ ^14^ ^,^ ^15^.

Além do PPA, foi resultado dessa ação civil pública a *Resolução Normativa n. 368*, de 6 de janeiro de 2015 ^16^, que garantiu o direito ao acesso à informação dos percentuais de cesáreas por estabelecimento de saúde no âmbito da saúde privada. Tais dados são disponibilizados nas bases de dados públicas do SINASC da Secretaria Municipal de Saúde de São Paulo ^17^.

Este artigo tem como objetivo avaliar a efetividade da intervenção de uma agência regulatória do governo brasileiro na redução das taxas de cesárea, avaliando a evolução das taxas por grupos de Robson das maternidades exclusivamente privadas da cidade de São Paulo que participaram desde a fase piloto do PPA, no período de 2014 (antes da implantação) a 2019 (quatro anos após a implantação). Os resultados serão comparados aos de maternidades exclusivamente privadas não participantes do projeto, no mesmo período e cidade. Será também descrita a taxa esperada de cesárea a partir da ferramenta *c-model* para o ano de 2019 para a população do estudo.

Em relação a outros estudos avaliativos do PPA, este estudo é inovador por comparar hospitais privados que não aderiram ao PPA com hospitais privados que aderiram desde o início do projeto, possibilitando melhor avaliação da diferença das taxas de cesárea entre os grupos e da efetividade do projeto.

## Métodos

Trata-se de estudo retrospectivo de base populacional que incluiu todos os nascimentos ocorridos de 2014 a 2019 de maternidades selecionadas a partir das bases de dados do SINASC, disponíveis no *site* da Secretaria Municipal de Saúde de São Paulo ^17^. A escolha por não considerar os anos posteriores a 2019 foi em razão da pandemia por COVID-19, que se iniciou em 2020 e poderia interferir nas taxas de cesárea.

Para avaliar a efetividade (resultado real na prática) da intervenção, foram criados dois grupos para comparação: um chamado de HPPA, composto pelas cinco maternidades da cidade de São Paulo que participaram desde a primeira fase do PPA ^14^, e outro chamado HNPPA, das 13 maternidades da cidade exclusivamente privadas que em nenhum momento participaram do programa do governo. Foram excluídas do estudo maternidades: públicas; privadas que com leitos públicos contratados; privadas que foram incluídas no PPA após sua primeira fase ^13^, e maternidades com menos de 500 partos no ano de 2019 ^17^.

**Figura 1:**
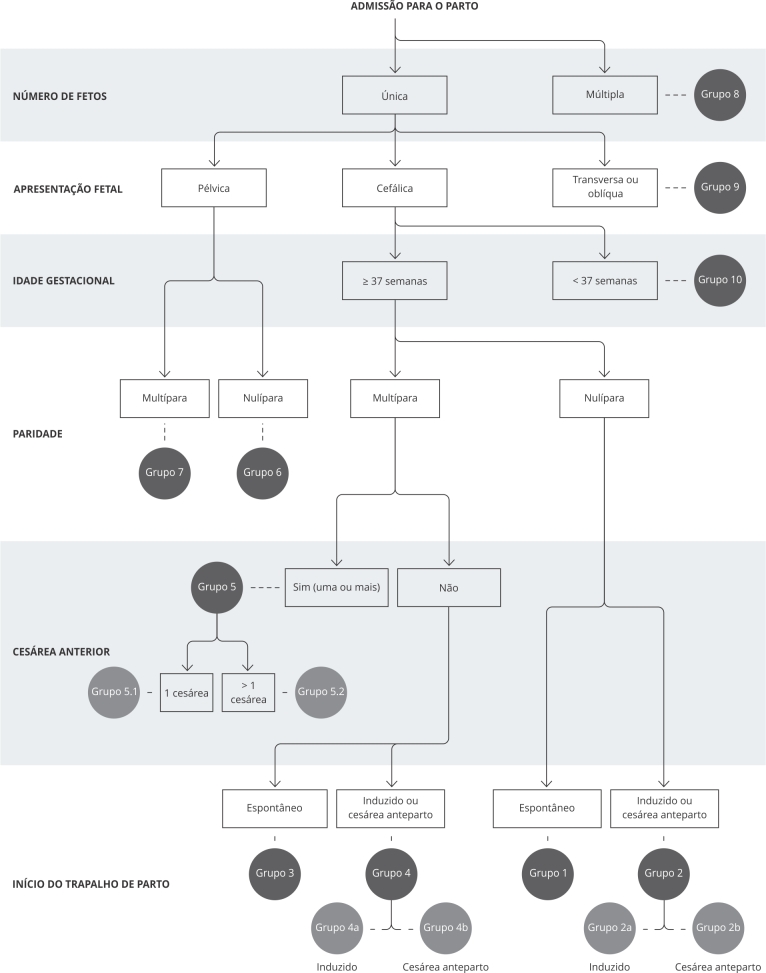
Classificação de Robson.

As mulheres foram classificadas por grupos de Robson (modificado de acordo com sugestão da OMS) ^18^ (Figura 1).

Fonte: Campos et al. ^25^.

As variáveis categóricas foram descritas com o uso de frequências absolutas e relativas ^19^. Os resultados das variáveis demográficas e maternas foram apresentados segundo a participação no PPA.

Para avaliar o efeito do PPA na probabilidade de cesárea, utilizaram-se modelos de regressão logística ajustados para variáveis demográficas e maternas, com efeito aleatório do hospital. Nesses modelos, foram inseridos termos de interação PPA * ano * Robson, para avaliar se o efeito do PPA dependia do ano e do grupo de Robson. Os *odds ratio* (OR) estimados pelo modelo foram apresentados em gráficos por ano e grupos de Robson com os respectivos IC95%.

O modelo foi construído da seguinte forma: tipo de parto ~ PPA * ano * Robson + idade materna + raça materna + estado civil + escolaridade + efeito aleatório do hospital. Assim, o ano foi utilizado como covariável de interação com o grupo PPA e Robson. Consequentemente, todas as variáveis ajustadas (maternas) foram estimadas em um único modelo, com controle das variáveis faixa etária, raça/cor, estado civil e escolaridade. A qualidade do modelo foi avaliada por um gráfico de calibração.

As análises foram realizadas com auxílio do software RStudio (https://rstudio.com/), versão 4.1.0. Para os testes de hipótese, considerou-se nível de 5% de significância.

Para o ano de 2019, os resultados dos dois grupos foram submetidos à ferramenta *c-model* para obter as taxas esperadas de cesáreas individualizadas para a população.

O banco de dados obtido será compartilhado por tempo indeterminado no repositório *Figshare* (https://figshare.com/s/c7f0619343cf9395c611), assim como o dicionário de dados e o *script* utilizado no RStudio.

Por serem dados secundários em domínio público, não foi necessária aprovação do Comitê de Ética em Pesquisa, conforme *Resolução nº 510*, de 7 de abril de 2016, do Conselho Nacional de Saúde.

## Resultados

Os dados utilizados abrangem a totalidade dos 277.747 nascidos vivos dos hospitais do estudo, no período de seis anos (2014 a 2019) ^20^. Apenas 1.390 (0,5%) casos não puderam ser classificados por grupos de Robson, e o tamanho relativo do grupo 9 foi de 0,2% com taxa de cesárea de 99% (esperado ser entre 0,2% e 0,6% com taxa de cesárea de 100%) o que denota a qualidade dos dados ^18^. A distribuição dos nascimentos por grupo (HPPA e HNPPA), de acordo com as características demográficas e obstétricas, está descrita na Tabela 1.

**Tabela 1:** Distribuição dos nascimentos de acordo com características demográficas e obstétricas e participação no Programa Parto Adequado (PPA). São Paulo, Brasil, 2014 a 2019.

Variáveis	Total		PPA - análise descritiva				Medida de efeito		
	n	%	Não (N = 146.530)		Sim (N = 131.217)		OR	IC95%	Valor de p *
			n	%	n	%			
Faixa etária (anos)									
< 20	11.481	4	9.265	6	2.216	2			
20-34	187.766	68	106.056	72	81.710	62	3,22	3,07-3,38	< 0,001
> 34	78.500	28	31.209	21	47.291	36	6,34	6,04-6,65	< 0,001
Raça/Cor								
Não branca	93.712	34	63.149	43	30.563	23			
Branca	183.899	66	83.280	57	100.619	77	2,50	2,46-2,54	< 0,001
Estado civil									
Sem união estável	93.090	34	62.813	43	30.277	23			
Com união estável	184.435	66	83.576	57	100.859	77	2,50	2,46-2,55	< 0,001
Escolaridade (anos de estudo) **									
< 12	119.631	43	89.894	61	29.737	23			
≥ 12	157.928	57	56.483	39	101.445	77	5,43	5,34-5,52	< 0,001
Cesárea prévia									
0	192.225	69	101.739	70	90.486	69			
1-2	83.373	30	43.340	30	40.033	31	1,04	1,02-1,06	< 0,001
> 2	1.661	1	965	1	696	1	0,81	0,74-0,89	< 0,001
Tipo de gravidez									
Única	268.503	97	143.338	98	125.165	95			
Múltipla	9.244	3	3.192	2	6.052	5	2,17	2,08-2,27	< 0,001
Paridade									
0	146.852	53	72.683	50	74.169	57			
1-2	95.572	34	50.849	35	44.723	34	0,86	0,85-0,88	< 0,001
> 2	34.829	13	22.509	15	12.320	9	0,54	0,52-0,55	< 0,001
Apresentação fetal									
Cefálica	263.625	95	140.597	97	123.028	94			
Pélvica	12.510	5	4.662	3	7.848	6	1,92	1,85-2,00	< 0,001
Transversa	566	0	240	0	326	0	1,55	1,31-1,84	< 0,001
Indução									
Não	240.333	87	127.157	87	113.176	86			
Sim	36.370	13	18.356	13	18.014	14	1,10	1,08-1,13	< 0,001
Cesárea antes do trabalho de parto									
Não	60.998	29	35.560	32	25.438	25			
Sim	151.990	71	75.910	68	76.080	75	1,4	1,37-1,43	< 0,001
Via de nascimento									
Vaginal	63.286	23	33.837	23	29.449	22			
Cesárea	214.459	77	112.691	77	101.768	78	1,04	1,02-1,06	< 0,001
Grupo de Robson									
1	37.629	14	19.697	14	17.932	14			
2	84.835	31	42.134	29	42.701	33	1,11	1,09-1,14	< 0,001
3	20.423	7	13.667	9	6.756	5	0,54	0,52-0,56	< 0,001
4	18.065	7	10.950	8	7.115	5	0,71	0,69-0,74	< 0,001
5	73.110	26	38.115	26	34.995	27	1,01	0,98-1,03	0,505
6	6.375	2	2.296	2	4.079	3	1,95	1,85-2,06	< 0,001
7	3.779	1	1.701	1	2.078	2	1,34	1,25-1,44	< 0,001
8	9.102	3	3.152	2	5.950	5	2,07	1,98-2,17	< 0,001
9	566	0	240	0	326	0	1,49	1,26-1,77	< 0,001
10	22.473	8	13.211	9	9.262	7	0,77	0,74-0,80	< 0,001

IC95%: intervalo de 95% de confiança; OR: *odds ratio*.

Fonte: Secretaria Municipal de Saúde ^17^.

* Teste de qui-quadrado de Pearson;

** 12 anos de estudo = Ensino Médio completo.

A Tabela 1 agrega as informações referentes ao período de 2014 a 2019 e mostra diferenças estatisticamente significantes nas características sociodemográficas: no grupo HPPA, a proporção de mulheres acima de 35 anos foi maior, e de mulheres abaixo de 20 anos, menor; no grupo HNPPA, a proporção de mulheres não brancas, assim como a de mulheres sem união estável, foi maior; chama a atenção a grande diferença na proporção de mulheres com menos de 12 anos de estudo nesse grupo. Em relação às características obstétricas, observamos uma proporção maior de nulíparas, assim como de gestações múltiplas e apresentação pélvica no HPPA. Outras diferenças, embora estatisticamente significantes, não se apresentam clinicamente relevantes.

A Tabela 2 mostra os parâmetros do modelo, com o controle das variáveis maternas faixa etária, raça/cor, estado civil e escolaridade.

**Tabela 2:** Efeitos estimados para cesárea, incluindo interação com grupos de Robson e hospitais do Programa Parto Adequado. São Paulo, Brasil, 2014 a 2019.

Variáveis	Estimativa	OR	IC95%	Valor de p
(Intercept)	1,32	3,74	2,45-5,72	0,0000
PPA - Sim	-0,28	0,75	0,33-1,71	0,4783
Ano - 2015	-0,38	0,68	0,63-0,74	0,0000
Ano - 2016	-0,23	0,79	0,73-0,85	0,0000
Ano - 2017	-0,19	0,82	0,76-0,89	0,0000
Ano - 2018	-0,29	0,75	0,69-0,81	0,0000
Ano - 2019	-0,14	0,87	0,80-0,94	0,0003
Robson 3+4	-1,55	0,21	0,19-0,23	0,0000
Robson 5	0,93	2,54	2,26-2,86	0,0000
Robson 6+7+9	1,79	5,99	4,11-8,74	0,0000
Robson 8	1,39	4,02	2,62-6,17	0,0000
Robson 10	-0,69	0,5	0,45-0,57	0,0000
Faixa etária (anos) - ≥ 20 e ≤ 34 *vs*. < 20	0,56	1,75	1,66-1,84	0,0000
Faixa etária (anos) - > 34 *vs*. < 20	0,91	2,49	2,35-2,63	0,0000
Raça/Cor - branca *vs*. não branca	0,02	1,02	1,00-1,05	0,0550
Estado civil da mãe - união estável *vs*. não	0,03	1,03	1,01-1,06	0,0168
Escolaridade da mãe - ≥ 12 anos *vs*. < 12 anos	-0,05	0,95	0,93-0,98	0,0009
PPA Sim: Ano - 2015	0,03	1,03	0,93-1,15	0,5218
PPA Sim: Ano - 2016	-0,20	0,82	0,74-0,92	0,0003
PPA Sim: Ano - 2017	-0,32	0,72	0,65-0,81	0,0000
PPA Sim: Ano - 2018	-0,45	0,64	0,57-0,71	0,0000
PPA Sim: Ano - 2019	-0,69	0,5	0,45-0,56	0,0000
PPA Sim: Robson 3+4	-0,31	0,73	0,64-0,84	0,0000
PPA Sim: Robson 5	0,50	1,66	1,38-1,99	0,0000
PPA Sim: Robson 6+7+9	-0,09	0,92	0,56-1,50	0,7314
PPA Sim: Robson 8	0,19	1,21	0,71-2,07	0,4897
PPA Sim: Robson 10	0,45	1,58	1,32-1,88	0,0000
Ano - 2015: Robson 3+4	0,10	1,1	0,97-1,25	0,1293
Ano - 2016: Robson 3+4	-0,06	0,94	0,83-1,07	0,3575
Ano - 2017: Robson 3+4	-0,04	0,96	0,84-1,09	0,5018
Ano - 2018: Robson 3+4	-0,03	0,98	0,85-1,11	0,7077
Ano - 2019: Robson 3+4	0,01	1,01	0,89-1,15	0,8707
Ano - 2015: Robson 5	0,37	1,45	1,23-1,70	0,0000
Ano - 2016: Robson 5	0,29	1,33	1,13-1,57	0,0008
Ano - 2017: Robson 5	0,23	1,26	1,07-1,49	0,0064
Ano - 2018: Robson 5	0,21	1,23	1,04-1,45	0,0136
Ano - 2019: Robson 5	0,27	1,3	1,10-1,54	0,0018
Ano - 2015: Robson 6+7+9	0,90	2,46	1,41-4,27	0,0015
Ano - 2016: Robson 6+7+9	0,54	1,72	0,98-3,03	0,0601
Ano - 2017: Robson 6+7+9	0,31	1,36	0,79-2,36	0,2655
Ano - 2018: Robson 6+7+9	-0,08	0,92	0,55-1,55	0,7564
Ano - 2019: Robson 6+7+9	0,37	1,45	0,84-2,52	0,1867
Ano - 2015: Robson 8	0,33	1,39	0,78-2,49	0,2622
Ano - 2016: Robson 8	0,54	1,72	0,87-3,42	0,1196
Ano - 2017: Robson 8	0,03	1,03	0,57-1,86	0,9284
Ano - 2018: Robson 8	-0,08	0,92	0,50-1,72	0,7982
Ano - 2019: Robson 8	-0,20	0,82	0,45-1,50	0,5133
Ano - 2015: Robson 10	0,28	1,32	1,12-1,55	0,0007
Ano - 2016: Robson 10	0,14	1,15	0,98-1,36	0,0960
Ano - 2017: Robson 10	0,08	1,08	0,91-1,29	0,3702
Ano - 2018: Robson 10	0,22	1,24	1,04-1,48	0,0152
Ano - 2019: Robson 10	0,05	1,05	0,88-1,25	0,5810
PPA Sim: Ano - 2015: Robson 3+4	0,03	1,03	0,85-1,24	0,7856
PPA Sim: Ano - 2016: Robson 3+4	0,18	1,19	0,98-1,45	0,0734
PPA Sim: Ano - 2017: Robson 3+4	0,07	1,07	0,88-1,30	0,4896
PPA Sim: Ano - 2018: Robson 3+4	0,18	1,19	0,98-1,45	0,0764
PPA Sim: Ano - 2019: Robson 3+4	0,12	1,13	0,93-1,37	0,2326
PPA Sim: Ano - 2015: Robson 5	-0,41	0,66	0,52-0,85	0,0010
PPA Sim: Ano - 2016: Robson 5	-0,46	0,63	0,50-0,81	0,0002
PPA Sim: Ano - 2017: Robson 5	-0,47	0,63	0,49-0,80	0,0001
PPA Sim: Ano - 2018: Robson 5	-0,41	0,66	0,52-0,84	0,0007
PPA Sim: Ano - 2019: Robson 5	-0,56	0,57	0,45-0,72	0,0000
PPA Sim: Ano - 2015: Robson 6+7+9	-0,58	0,56	0,28-1,15	0,1128
PPA Sim: Ano - 2016: Robson 6+7+9	-0,04	0,96	0,46-1,99	0,9167
PPA Sim: Ano - 2017: Robson 6+7+9	0,59	1,81	0,87-3,78	0,1135
PPA Sim: Ano - 2018: Robson 6+7+9	1,35	3,86	1,81-8,20	0,0005
PPA Sim: Ano - 2019: Robson 6+7+9	0,57	1,76	0,84-3,70	0,1331
PPA Sim: Ano - 2015: Robson 8	0,01	1,01	0,49-2,10	0,9806
PPA Sim: Ano - 2016: Robson 8	-0,41	0,66	0,30-1,49	0,3197
PPA Sim: Ano - 2017: Robson 8	0,39	1,47	0,70-3,09	0,3080
PPA Sim: Ano - 2018: Robson 8	0,8	2,21	1,03-4,78	0,0428
PPA Sim: Ano - 2019: Robson 8	0,38	1,47	0,70-3,06	0,3067
PPA Sim: Ano - 2015: Robson 10	-0,26	0,77	0,60-0,98	0,0347
PPA Sim: Ano - 2016: Robson 10	-0,08	0,93	0,72-1,19	0,5462
PPA Sim: Ano - 2017: Robson 10	0,16	1,17	0,91-1,51	0,2121
PPA Sim: Ano - 2018: Robson 10	0,06	1,06	0,82-1,36	0,6574
PPA Sim: Ano - 2019: Robson 10	0,35	1,42	1,10-1,83	0,0075

IC95%: intervalo de 95% de confiança; OR: *odds ratio*.

Fonte: Secretaria Municipal de Saúde ^17^.

**Figura 2:**
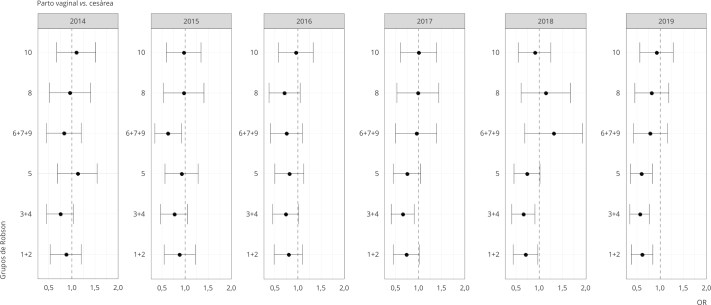
*Odds ratio* (OR) para cesárea para hospitais participantes e não participantes do Programa Parto Adequado (PPA) por grupos de Robson por ano. São Paulo, Brasil, 2014 a 2019.

Em 2014, a taxa de cesáreas dos HPPA era de 83,8%, enquanti a dos HNPPA era de 78,9%. Jpa em 2019, nos HPPA, a taxa de cesárea foi de 72,3% e, nos HNPPA, 76,2%. A Figura 2 apresenta a estimativa de OR, com seu respectivo IC95%, para cada ano, do efeito do PPA na taxa de cesárea (usando como variável binária o tipo de parto), considerando a interação com o ano, ajustada por variáveis maternas e grupos de Robson. Observamos que todos os efeitos de interação entre ano e participação no PPA foram significativos na chance de cesárea. Isso indica que o efeito do PPA depende do ano analisado e, portanto, deve ser estimado para cada ano.

Fonte: Secretaria Municipal de Saúde ^17^.

Não foi observado efeito do PPA na redução da chance de cesárea em 2014, ou seja, antes do lançamento do programa, mas notou-se uma tendência constante de redução, até se tornar significativa em 2018. Esse resultado ocorreu de forma independente das variáveis demográficas, maternas e dos grupos de Robson. Em 2014, a redução na chance de cesárea para os participantes do PPA não foi significativa para nenhum dos grupos de Robson. Observa-se um efeito protetor significativo do PPA, com redução na chance de cesárea para os grupos 3 e 4, em 2017; a partir de 2018, para os grupos de 1 a 4; e, em 2019, para os grupos de 1 a 5.

Outros resultados observados no modelo foram uma chance de cesárea maior nas faixas etárias mais elevadas em relação às abaixo de 20 anos, tendo sido de 1,73 entre as mulheres de 20-34 anos e 2,44 nas com mais de 34 anos. Mulheres brancas e com união estável apresentaram uma chance de cesárea 4% e 3% maiores, respectivamente, enquanto as com 12 anos ou mais de estudo apresentaram chance 4% menor de cesárea em relação àquelas com menor escolaridade.

As estimativas do modelo para taxa de cesárea considerando a interação tripla do PPA com os grupos de Robson e ano mostrou que há efeito de interação significativo, logo os efeitos médios do PPA ajustados por variáveis maternas devem ser estimados para cada ano e para cada grupo de Robson, assim como apresentado na Figura 2.

**Figura 3:**
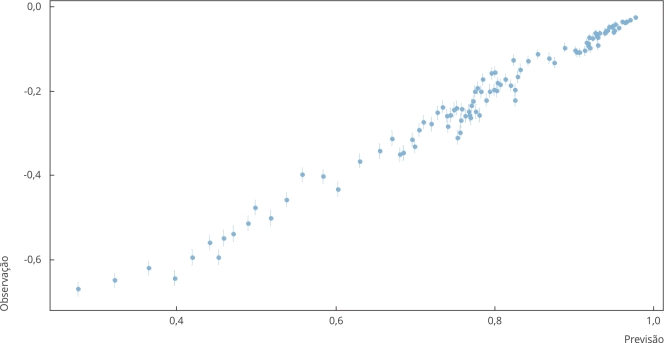
Gráfico de calibração do modelo utilizado.

A Figura 3 mostra o gráfico de calibração, utilizado para a avaliação da qualidade do modelo.

Para avaliar desfechos neonatais, o Apgar foi estudado de forma binária como sendo < 7 ou ≥ 7. Essa variável foi avaliada no tempo e ajustada por variáveis maternas e participação no PPA. O objetivo principal foi avaliar se houve redução do Apgar ao longo do estudo em função de um possível efeito negativo do PPA. Observamos que não houve aumento da chance do Apgar < 7 com o PPA. Não identificamos mudanças do Apgar em relação ao tempo, à faixa etária, à cor/raça, à escolaridade materna. Nesse modelo (dados não mostrados), a chance de Apgar < 7 na cesariana foi três vezes a chance do Apgar < 7 no parto vaginal.

Fonte: Secretaria Municipal de Saúde ^17^.

Para o ano de 2019, foi observada uma taxa de cesárea de 72,3% (IC95%: 71,7-72,9) no grupo do PPA, bem acima da taxa esperada de acordo com a ferramenta *c-model* que foi de 45,2% (IC95%: 33,9-56,5) ^13^.

## Discussão

O PPA foi o primeiro programa sistemático de melhoria na qualidade da assistência obstétrica implementado no setor privado, tendo sido também o primeiro a ter um impacto para a redução de cesáreas desnecessárias. Sua criação foi uma das decorrências da ação civil pública no Estado de São Paulo, com repercussões nacionais, movida pela rede Parto do Princípio, perante as altas taxas de cesárea praticadas nesse setor, que atinge patamares acima de 80% ^21^ ^,^ ^22^. A rede apresentou, em 2006, um dossiê ao Ministério Público Federal denunciando as altas taxas de cesárea e propondo mudanças no setor privado. Em 2010, o Ministério Público Federal ajuizou a Ação Civil Pública contra a Agência Nacional de Saúde Suplementar (ANS) exigindo a regulamentação da assistência obstétrica, em atenção às propostas do dossiê da rede Parto Princípio. Com essa ação, a ANS propôs, em 2015, a criação do PPA ^21^.

O modelo de atenção ao parto no setor privado é centrado na figura do médico, responsável pela condução do pré-natal, parto e puerpério mesmo das mulheres de risco habitual, sem a participação efetiva de enfermeiras obstétricas/obstetrizes. Tais profissionais atuam com autonomia sobre as condutas tomadas, nem sempre seguindo os protocolos assistenciais baseados em evidências científicas e no uso criterioso das intervenções clínicas quando necessárias. Nesse sentido, o PPA foi criado para promover um novo modelo de atenção ao parto, estimulando o parto espontâneo por meio da qualificação da assistência e de intervenções no processo assistencial ^21^ ^,^ ^22^.

No decorrer dos anos avaliados, ocorreu uma redução da taxa geral de cesáreas entre os hospitais participantes e não participantes do PPA. Tal redução pode estar relacionada à disseminação entre as mulheres e os profissionais de práticas assistenciais e recomendações baseadas em evidências científicas, como planos de parto, presença de acompanhantes e mudanças na ambiência.

Porém, essa redução foi mais acentuada entre os hospitais que participaram do PPA, particularmente nos grupos 1 a 5 de Robson, evidenciando que o apoio institucional, científico e metodológico do PPA aos hospitais que desejaram reorganizar seu modelo de atenção ao parto e ao nascimento promovido foi uma medida efetiva para reduzir cesáreas injustificadas clinicamente.

Outros estudos que avaliaram o PPA corroboraram que foi possível reduzir a prevalência de cesáreas em hospitais privados no Brasil, além de uma redução nos desfechos negativos maternos e neonatais e uma relação custo-efetividade maior ^23^. Tal redução ocorreu principalmente nas cesáreas sem indicação clínica, que compõem grande parte do excesso praticado. O sucesso do projeto foi uma consequência do engajamento das lideranças do setor saúde, da participação das mulheres e famílias, da reorganização dos modelos de atenção ao parto e do monitoramento dos resultados ^22^ ^,^ ^24^. Porém, somente o engajamento de mulheres não se mostrou eficaz para tal redução, tendo sido necessária a participação das instituições, principalmente privadas, que foram motivadas a participar devido à ausência de outros programas para melhoria na qualidade, a partir de uma recomendação da ANS ^21^.

Os resultados deste estudo mostraram que uma política pública bem conduzida com o envolvimento de instituições privadas pode mudar o cenário da atenção ao parto e ao nascimento, promovendo a redução das altas taxas de cesárea. Porém, tais taxas ainda permanecem além do esperado para a população de estudo, de acordo com a ferramenta *c-model* referenciada pela OMS.
